# Structure–function comparison of Arbekacin with other aminoglycosides elucidates its higher potency as bacterial translation inhibitor

**DOI:** 10.1038/s41598-025-02391-3

**Published:** 2025-05-25

**Authors:** Soneya Majumdar, Narayan Prasad Parajuli, Xueliang Ge, Suparna Sanyal

**Affiliations:** Department of Cell and Molecular Biology, Biomedical Center, Uppsala University, Uppsala, 75124 Sweden

**Keywords:** Cryoelectron microscopy, Cryoelectron microscopy, Biophysical chemistry

## Abstract

Aminoglycoside antibiotics are well-known inhibitors of bacterial protein synthesis, which act mainly by inhibiting ribosomal translocation and inducing miscoding errors. Arbekacin (ABK) is a semisynthetic aminoglycoside that was developed by adding a 3-amino-2-hydroxybutyric (AHB) moiety to the 2-deoxystreptamine (2-DOS) ring of dibekacin for counteracting the problem of enzyme-mediated resistance of aminoglycosides. Here, we have systematically compared the inhibition efficacy of ABK with other aminoglycosides by in vivo MIC determination and in vitro fast-kinetics based translocation and termination assays complemented with a high-resolution cryo-EM structure. ABK presents significantly lower MIC_50_ value compared to its parent antibiotics kanamycin and dibekacin. Consistent with that, ABK inhibits translocation with lower inhibition constant and reside on the ribosome for significantly longer time than the classical aminoglycosides. Our 3.1 Å resolution cryo-EM structure of ABK-bound ribosome containing mRNA and initiator-tRNA, shows interactions of the unique AHB moiety of ABK with rRNA nucleobases, which likely provide additional stabilization of ABK at the canonical aminoglycoside binding pocket and contribute to its prolonged dwelling time. Our structural and functional analyses provide molecular basis for higher potency of ABK in bacterial translation inhibition and opens the possibility of rational design of new antibiotics.

**Supplementary Information:**

The online version contains supplementary material available at 10.1038/s41598-025-02391-3.

## Introduction

The ribosome is an attractive antibiotic target in bacterial cells. Many clinically successful antibiotics inhibit protein synthesis in bacteria by binding to the functional centers of the ribosome and trapping it to a particular conformation or hindering its interactions with the ligands^1,2^. The ribosome-targeting antibiotics, therefore, are indispensable tools for therapeutic purposes, and also, for mechanistic understanding of ribosome functions^3^. Aminoglycosides (AGAs) are among the most successful antibiotics in treating bacterial infections since their discovery in the 1940s. AGAs inhibit bacterial protein synthesis by inhibiting EF-G mediated tRNA translocation, RF1/RF2 mediated peptide release and by inducing miscoding errors during decoding the genetic information^4–12^.

**Fig. 1:**
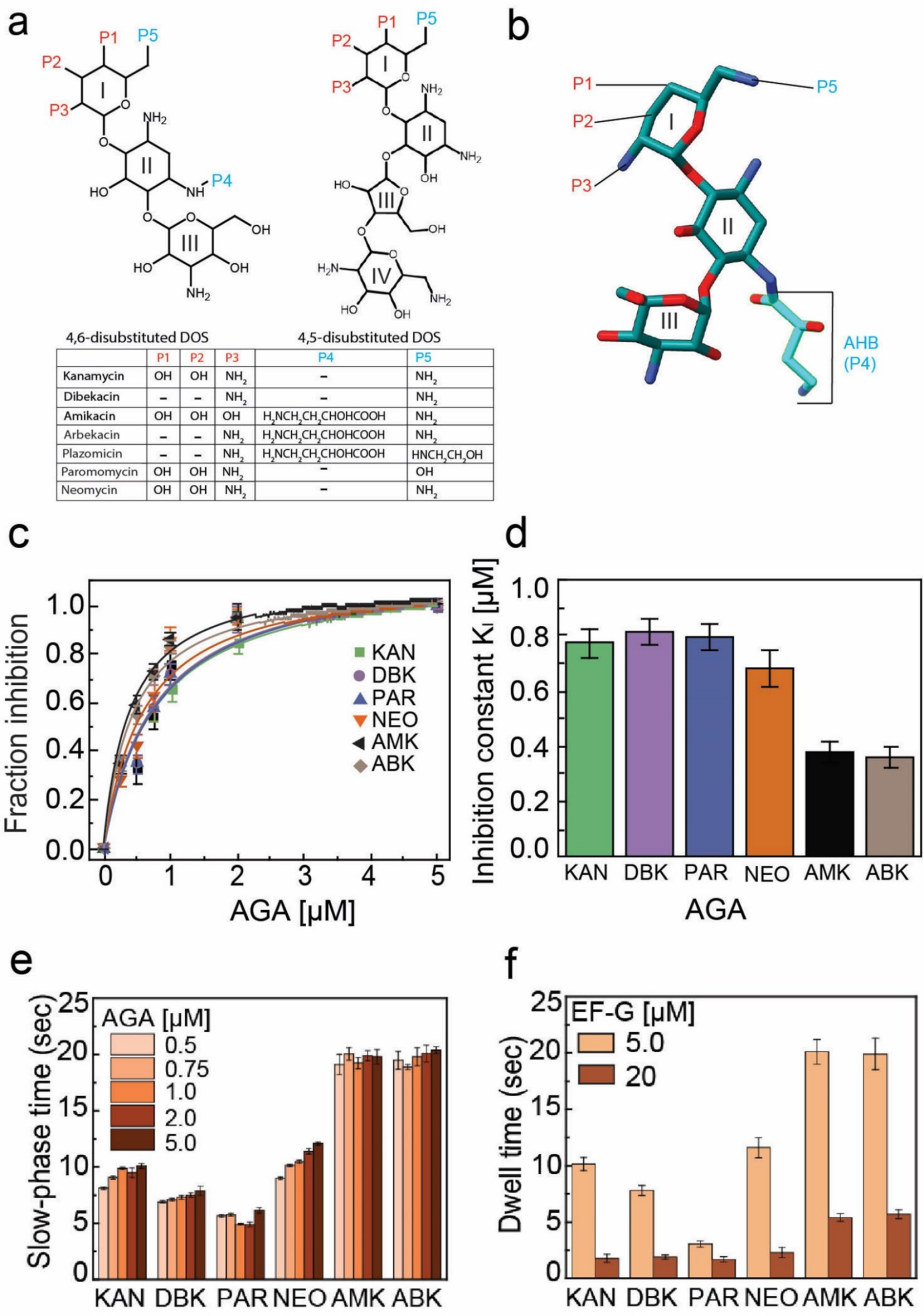
Systematic comparison of the AGAs for their effectiveness in inhibition of tRNA-mRNA translocation. (**a**) Comparison of chemical structures of the 4,6-disubstituted DOS and 4,5-disubstituted DOS AGAs. (**b**) Structure of Arbekacin (ABK) showing the AHB3-amino-2-hydroxybutyric (AHB) tail (cyan). ABK is synthesized by adding the AHB moiety to Dibekacin (DBK) (Supplementary Fig. 1). (**c**) The fraction of AGA-inhibited ribosomes with various AGAs as estimated from the translocation inhibition assay (Experimental data in Supplementary Fig. 2). Solid lines represent the hyperbolic fit of the fraction inhibited, estimated from the slow phase amplitude of the translocation kinetics fluorescence traces (Supplementary Fig. 2) in the presence of various concentration of the respective AGAs. (**d**) Inhibition constants (K_I_) of the various aminoglycosides on the inhibition of translocation. Experiments were conducted in triplicates and error bars indicate the SEM of data. (**e**) Concentration-dependent effect of the AGAs on pyrene-mRNA movement during ribosomal translocation monitored in stopped-flow (Supplementary Fig. 2). The fluorescent traces were fitted with double exponential function and the slow-phase time was estimated by using the translocation inhibition model published in our earlier study^11^. (**f**) The dwell-time of various AGAs on pre-translocation ribosomes with 5 µM of EF-G and 20 µM of EF-G estimated from the slow-phase time as in^11^. Data points represent an average of at least three replicates with indicated SEM.

There are four structural groups of AGAs of which three have an aminocyclitol 2-deoxystreptamine (2-DOS) ring, also referred to as ring-II (R-II)^13,14^ (Fig. 1a). The AGA subclasses are based on the substitutions on R-II—4,5-disubstituted (neomycin and paromomycin) or 4,6-disubstituted (kanamycin) having ring-I (R-I) and ring-III (R-III) substitutions at 4,5 or 4,6 positions of R-II^15^ (Fig. 1a). All 2-DOS AGAs bind to a common binding pocket on bacterial ribosome at helix 44 (h44) of 16S rRNA near the decoding center (DC) at the A site of the 30S subunit^15,16^. The R-I and R-II rings interact with conserved G1491, G1498 and A1408 rRNA bases (*Escherichia coli* numbers) on 16S rRNA. Binding of AGAs to the ribosome pushes two conserved monitoring bases on h44, namely A1492 and A1493, out of the helix. As a result, they acquire a flipped-out conformation similar to that when a cognate tRNA is accepted in the A-site. This conformational change likely induces error in protein synthesis by allowing incorrect tRNAs to decode, as well as causes inhibition of translocation and termination. In addition to this, some AGAs neomycin (NEO), paromomycin (PAR) and amikacin (AMK) also bind to the H69 of 23S rRNA and are suggested to alter ribosome dynamics during translocation and ribosome recycling^7,9,14,17–20^.

Although AGAs present strong arsenal against bacterial infections, AGA modifying enzymes (AMEs)-mediated resistance limits their effectivity against bacterial pathogens^21^. These enzymes usually modify different side chains of the R-I, R-II and R-III rings causing loss of affinity of the AGAs to the ribosome and rendering them ineffective. Arbekacin (ABK) (Fig. 1b) is a new-generation semisynthetic AGA that was originally developed to overcome the problem of AME-mediated resistance of AGAs^21^. It is approved for the treatment of multiple drug-resistant (MDR) pneumonia and septicemia in many countries, including Japan, Korea, India, and the United States^16,22^. Chemically, ABK, a derivative of kanamycin B (KAN), belongs to the 4,6-disubstituted 2-DOS sub-class of AGAs with a 3-amino-2-hydroxybutyric (AHB) moiety attached at the N^1^ position (P4, Fig. 1a) of the 2-DOS ring (R-II) (Fig. 1a, B)^23^. Dibekacin (DBK) is the parental compound of ABK, which lacks the two OH groups that are target of the AGA modifying enzymes (Fig. 1a). ABK is synthesized by adding the AHB moiety to DBK (Supplementary Fig. 1). Due to the absence of OH groups at 3′ (P2) and 4′ (P1) positions of R-I, ABK and DBK are protected against the activity of AMEs, AGA nucleotidyl-transferase (ANT) (4′) and AGA phosphotransferase (APH) (3′)^22,24^. Moreover, due to addition of the AHB moiety at the N^1^ position (P4, Fig. 1a) of R-II, ABK is impervious to the action of AGA acetyl-transferase (AAC) (3′, RI), ANT (2′′, R-III), and APH (2′′, R-III), the most common AMEs in Enterobacteriaceae^22,25^. Curiously, despite being acetylated by AMEs including members of the AAC(6′)-I family and the bifunctional enzyme AAC(6′)/APH(2′′) reported in *S. aureus* and Enterococci^26,27^, ABK still retains strong antibiotic activity^22,28^.

Other than ABK, there are two AHB containing semisynthetic AGAs: (i) Amikacin (AMK) that is derived from kanamycin A by addition of AHB group. The difference of AMK from ABK is that AMK still retains the two OH groups at 3′ (P2) and 4′ (P1) positions of R-I similar to KAN unlike ABK (Fig. 1a). (ii) Plazomicin (PLA), which is derived from sisomicin with an AHB substitution at N^1^ position (P4, Fig. 1a) of R-II and a hydroxyethyl group at N^6^ position (P5, Fig. 1a) of R-I. Due to the later substitution the drug is resistant to deactivation by the most prevalent AAC(6′)-Ib.

We have recently clarified the comprehensive mechanism of ABK inhibition of bacterial protein synthesis demonstrating that ABK is a strong inhibitor of tRNA-mRNA translocation and translation termination, and that it severely dampens the accuracy of mRNA translation^11^. In another recent work, we have established the structural and biochemical basis of AMK inhibition of bacterial translation^29^. Recent studies have revealed that the AHB moiety of the antibiotics AMK, PLA and ABK engages in extensive interactions with rRNA nucleotides, that are absent in classical AGAs lacking this moiety^29–32^. Notable that the conformation of R-I in PLA is subtly different from that of other ribosome-bound aminoglycosides due to its hydroxyethyl group, which interacts not only with A1408 and G1494 of the 16S rRNA but also with A1913 of the 23S rRNA^32^. However, the precise impact of these interactions on the relative potency of the AHB-containing AGAs, specifically ABK, compared to other AGAs remained unexplored. Also, a comprehensive structural comparison between ABK, AMK and the classical AGAs is lacking in the literature.

In this work, we present a quantitative comparison of ABK with other AGAs in inhibiting two major steps of bacterial protein synthesis. In parallel to antibiotic susceptibility test and MIC_50_ measurements^33^ we have studied the effect of ABK and other antibiotics in inhibiting (i) translocation in elongating ribosomes, and (ii) class-I RF-mediated nascent peptide release in fully standardized fast-kinetics assays for translocation^34^ and termination^35^ by using a fully reconstituted in vitro translation system^36^, composed of purified translation components from *E. coli*. The tested AGAs include both new generation AGAs with AHB tail ABK and AMK and classical AGAs (without AHB moiety) including 4,5 DOS such as NEO, PAR and 4,6 DOS such as KAN, and DBK. We could not include PLA in our experiments due to unavailability of the drug. Our results of fast-kinetics experiments of translocation and termination clearly demonstrate ABK and AMK have much higher potency of inhibition of bacterial translation than other AGAs. To understand the molecular basis of high-potency inhibition by ABK we have solved a cryo-EM structure of ABK-bound 70S ribosome containing mRNA and initiator-tRNA. Our 3.1 Å resolution structure demonstrates additional interactions of AHB moiety of ABK to the 16S rRNA nucleobases upstream to the decoding center similar to the recent studies^29,31^. In addition, we report detailed structural analysis of AGA interactions with the ribosome and comparison with other AGAs, which provides the molecular basis for the higher potency of ABK and other AHB moiety-containing AGAs.

## Results

### The semi-synthetic AGAs, ABK and AMK, show much lower MIC values than the parental AGAs

We compared the minimum inhibitory concentrations (MIC) of ABK, AMK, DBK and KAN for *E. coli* MG1655 using the standard broth microdilution method. Our results indicate that of MICs of KAN for (≥8 µg/mL) and DBK (≥6 µg/mL) were high compared to MICs of ABK (~1 µg/mL) and AMK (~1 µg/mL). The lower MIC value for ABK and AMK demonstrate their higher efficacy in inhibition of bacterial growth and might origin from their higher affinity for the primary binding site.

### Higher inhibitory effect of AHB-containing AGAs, ABK and AMK, over classical AGAs on EF-G catalyzed tRNA translocation

To study the relative effects of various AGAs on the EF-G catalyzed mRNA translocation, we deployed both classic (NEO, PAR, KAN, and DBK) and semi-synthetic AHB-containing AGAs (ABK and AMK) in the pyrene-mRNA-based translocation assay^34^. As in our earlier work^11^, an ‘initiation mix’ containing 70S ribosomes programmed with 3’-pyrene labeled mRNA coding for Met-Phe-Leu and carrying fMet-tRNA^fMet^ in the P-site was rapidly mixed with an ‘elongation mix’ of ternary complex EF-Tu•GTP•Phe-tRNA^Phe^ and EF-G in a stopped-flow instrument. In this experiment, the movement of pyrene-labeled mRNA during translocation results in a time-dependent fluorescence change, thereby allowing real-time measurement of EF-G catalyzed mRNA movement^34^. Comparison of fluorescence traces recorded in the absence and presence of various AGAs in the ‘initiation mix’ signifies the relative inhibition of mRNA-tRNA translocation on aminoglycoside-bound ribosomes. Similar to ABK^11^, all AGAs produced biphasic fluorescence traces, where the amplitudes of the slow phase increase with the rising concentration of AGAs (Supplementary Fig. 2). The relative amplitudes of the slow phases reflecting inhibited fraction of the translocating ribosome increased hyperbolically with aminoglycoside concentrations (Fig. 1c), allowing us to determine precisely the half-maximal inhibitory concentrations (K_I_) for each AGA (Fig. 1d). The K_I_ values for ABK and AMK were almost 50% smaller compared to the classic AGAs including DBK and KAN, which are precursor AGAs for ABK and AMK, respectively (Fig. 1d). These results suggest that AHB-containing AGAs have at least two-fold higher affinity to the pre-translocation ribosomes than classic AGAs.

### The AHB-containing AGAs, ABK and AMK, dwell longer than classical AGAs on the ribosome

During the translocation inhibition assay (Supplementary Fig. 2), we observed that despite the increase in the concentration of aminoglycosides, the slow phase rate of the fluorescence traces remains constant (Fig. 1e), irrespective of whether the AGAs were pre-incubated with the ribosomes or added only in the elongation mix. Thus, the average times of the slow phases for each AGA would reflect how long the AGA resides on the ribosome. We have thus denoted the mean time of the slow phase as ‘dwell time’ for each AGA on the pre-translocation ribosome (Fig. 1e, f) as reported earlier^37^. At 5 µM EF-G, the average times of mRNA movement on ribosomes bound to classic aminoglycosides were 9.3 ± 0.6, 9.8 ± 0.8, 6.5 ± 0.5, and 8.2 ± 0.6 s for KAN, NEO, PAR, and DBK respectively (Fig. 1f). However, for ABK and AMK, this average dwell time was much longer i.e., 19.7 ± 1.6 and 20.3 ± 2.2 s, respectively (Fig. 1f). Next, we estimated the dwell times for each AGAs on the ribosomes by increasing the EF-G concentration from 5 to 20 µM. The dwell time of the AGAs, both classic and AHB-containing AGAs, decreased with higher EF-G concentration, suggesting a similar mechanism reported earlier for ABK^11^. However, the dwell time of ABK and AMK was still significantly longer than KAN, NEO, PAR, and DBK (Fig. 1f). Analyzing the inhibition constants and dwell times of DBK with other AGAs including the classical and the semisynthetic ABK and AMK, we conclude that the presence of the AHB moiety provides enhanced stability of these AGAs on the ribosome.

### Effect of ABK and other AGAs on RF-mediated peptide release

**Fig. 2:**
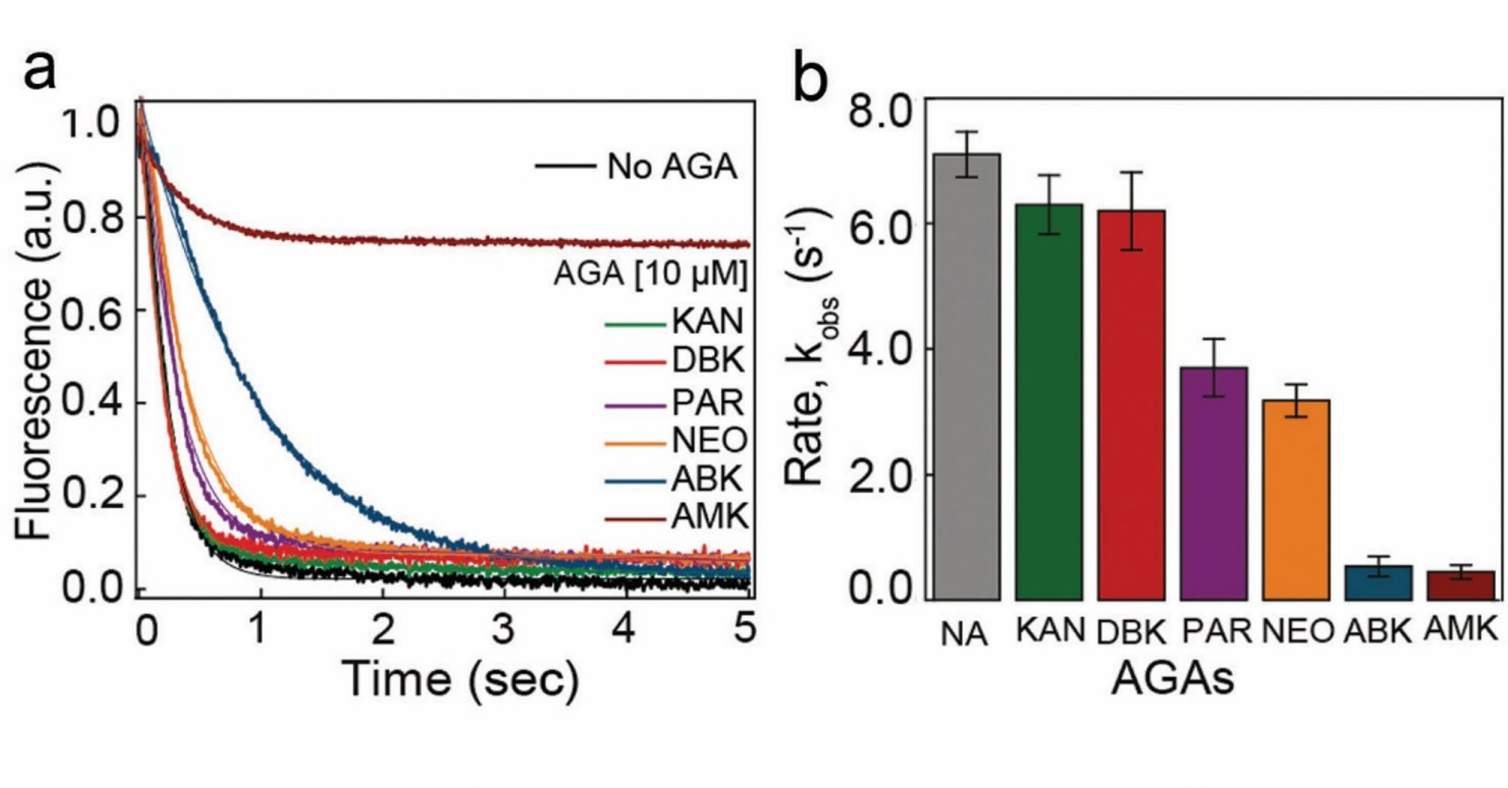
Effect of various AGAs on RF mediated translation termination. (**a**) Real-time BODIPY 576/589 (BOP) fluorescence traces for the release of (BOP)-Met-Phe-Leu tripeptide from the ribosome by RF2 (1 µM) without or with AGAs (10 µM). The curves were fitted with double exponential function and the rates were estimated from the inverse of average times of the fast phase. (**b**) Rates of peptide release in the absence and presence of various AGAs. The data represents an average of at least three replicates with indicated SEM.

AGA binding to the DC may impose a potential hurdle for class-I release factors (RF) to recognize stop codons during translation termination^11,38^. We sought to investigate the comparative effects of various AGAs on RF-mediated termination of translation in bacteria. For that, we prepared functional pre-termination ribosome complexes (pre-TC) with a peptidyl tRNA^Leu^ carrying fluorescent BODIPY 576/589 (BOP) labeled Met-Phe-Leu tripeptide in the P site, and an mRNA coding Met-Phe-Leu and stop codon (UAA) in the A site. The BOP-labelled fMet-Phe-Leu tripeptide was formed using a fully reconstituted translation system as described earlier^35^. The pre-TC was rapidly mixed with the RF mix containing an excess of RF2 using a stopped-flow instrument. The release of the (BOP)-Met-Phe-Leu tripeptide resulted in a nearly monophasic fluorescence decrease with time signifying the single-turnover peptide release (Fig. 2a). In the absence of AGAs, the apparent rate (*k*_obs_) of tripeptide release from the pre-TC was 7.1 ± 0.8 s^− 1^ (Fig. 2b). When similar experiments were conducted with the addition of various AGAs to both pre-TC and RF mixes, varied results were obtained. Among classical AGAs, KAN and DBK did not show any noticeable inhibition in peptide release (Fig. 2a, b). However, PAR and NEO reduced the rate of peptide release (*k*_obs_) to ≈ 3.6 ± 0.4 s^− 1^ and 3.2 ± 0.3 s^− 1^, respectively (Fig. 2b). Notably, ABK and AMK showed the most pronounced effect in the rates of peptide release (0.53 ± 0.09 s^− 1^^1^ with ABK and 0.45 ± 0.05 s^− 1^ with AMK) indicating more than 10-fold reduction in the rate of peptide release compared to no AGAs (Fig. 2b). In addition, AMK produced a significant reduction in the amplitudes of fluorescence traces indicating a unique effect of AMK on translation termination^29^.

### Structural analysis of *E. coli* 70S ribosome in complex with mRNA, fMet-tRNA^fMet,^ and ABK with cryo-EM

**Fig. 3:**
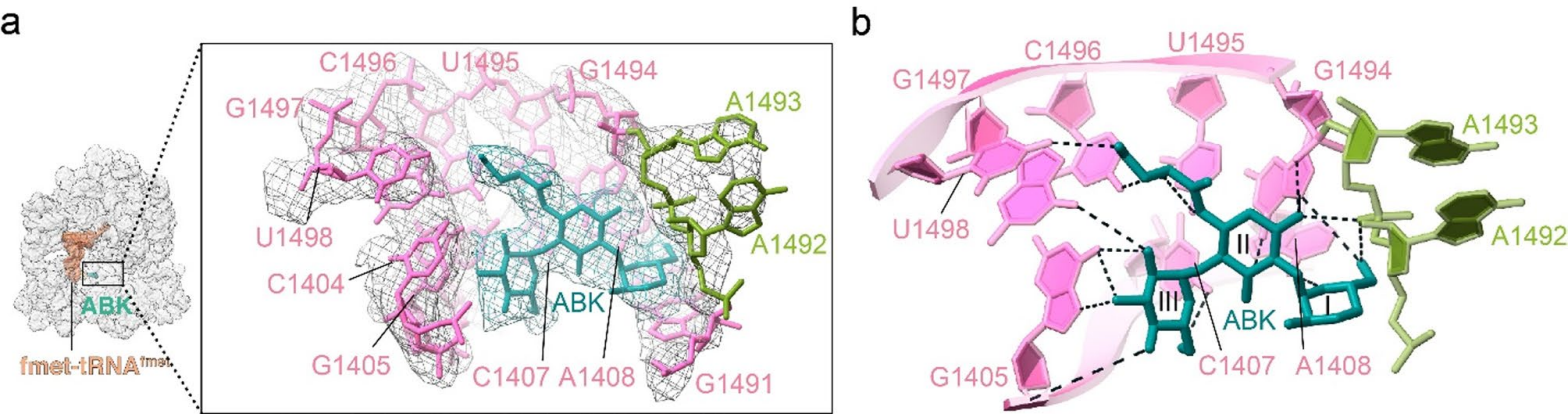
Structure of *E. coli* 70S ribosome-fMet-tRNA^fMet^-arbekacin complex. (**a**) EM density map of the *E. coli* 70S ribosome-fMet-tRNA^fMet^ complex bound to ABK in the primary aminoglycoside binding site; fMet-tRNA^fMet^ (salmon) and ABK (ABK, teal). The zoomed inset presents EM density and atomic model of ABK (teal) and the A-site rRNA (pink) nucleotides interacting with it. The h44 monitoring bases A1492 and A1493 are shown in olive green. (**b**) Molecular interactions stabilizing ABK at the primary binding site. The structure has been submitted in the database with PDB ID 9MKK.

We have solved the cryo-EM structure of functional *E. coli* 70S ribosome in complex with mRNA, fMet-tRNA^fMet,^ and ABK at 3.1 Å resolution (Fig. 3) (PDB:9MKK). Clear density was observed for almost all ribosomal proteins except bS1 and bL12, which are known to be extended and flexible. We could unambiguously model most of the rRNA, P-site bound fMet-tRNA^fMet,^ and the mRNA (Fig. 3a). Further examination of the cryo-EM map of the complex shows a clear density of ABK in the major groove of 16S rRNA at h44 near the decoding center (Fig. 3a). This site corresponds to the canonical binding site of the 2-DOS aminoglycosides^18,19^. Interestingly, the second binding site reported for certain aminoglycosides at H69 of 23S rRNA is missing in our structure^9^. Additionally, although an alternate binding site for ABK near the CCA end of the P-tRNA was reported previously^31^, we observe very weak density for the drug at this site in our structure (Supplementary Fig. 3), likely indicating that the affinity of this secondary site for the drug is weaker than that of the primary site.

### Comparison of ABK binding at the primary binding site

The cryo-EM structure allowed us to map specific interactions stabilizing ABK at the AGA binding pocket of h44 (Fig. 3b, Supplementary Table 1). The N3 amino group of the deoxystreptamine (DOS) ring (R-II) interacts with G1494 and the sugar-phosphate backbone of the flipped base A1493 while the O5 hydroxyl of RII interacts with the nucleotide G1494. The (S)-4-amino-2-hydroxybutyl (AHB) side chain in the 1-amino position of R-II interacts with the neighboring nucleotides, U1495, C1496 and U1498. R-I of ABK stacks with the base G1491, which appropriately positions it to interact with A1408. Further, R-III of ABK hydrogen bonds with the Hoogsteen sites, O6 and N7 of G1405 as well as N4 and O6 of C1407 and C1497 respectively. As we neared the completion of our work, a structure of *E. coli* 70S-ABK bound to A and P tRNA was reported (PDB: 8IFC)^31^. Comparison of the primary ABK binding site in our structure with the above structure of *E. coli* 70S-ABK^31^ shows a comparable conformation of ABK in these two structures (Supplementary Fig. 4).

### Structure based comparison of rRNA interactions of ABK and other AGAs in the primary binding site

**Fig. 4:**
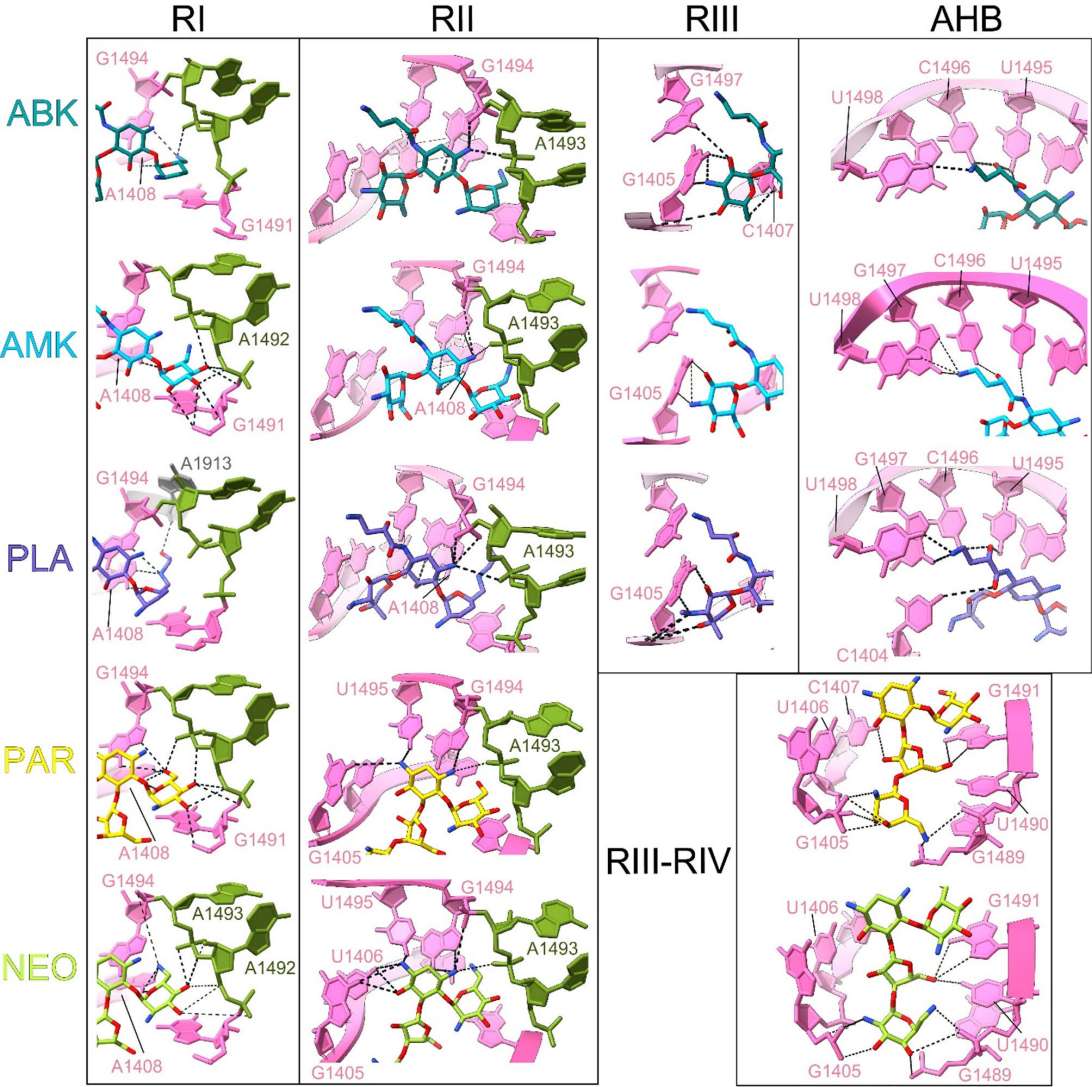
Structure based comparison of interactions of ABK, AMK, PLA, PAR, and NEO at the h44 AGA binding pocket. Detailed analysis of the interactions of the individual rings (RI-RIII/RIV) and AHB extension of the selected AGAs; ABK (teal), AMK (cyan), PLA (purple), PAR (yellow) and NEO (light green) with the h44 rRNA bases (in pink) are shown. The monitoring bases A1492 and A1493 are in olive green. The comparison is based on the following PDBs: ABK (PDB:9MKK, this study), AMK (PDB:8SYL)^29^, PLA (PDB:7LH5)^32^, NEO^9^ (PDB:4V52) and PAR (PDB:7K00)^39^.

ABK reveals a similar binding pattern at h44 of 16S rRNA as the other 4,6-substituted AGA, AMK (PDB:8SYL)^29^ and PLA (7LH5)^32^, and two 4,5- substituted AGAs NEO^9^ (PDB:4V52) and PAR (PDB:7K00)^39^. A comparison of our structure with the previously published structures (Fig. 4) shows that the DOS-ring is held by a set of conserved interactions from nucleotides A1408 and, G1494. A few drug specific interactions are also seen as elaborated below (Fig. 4) and summarized in Supplementary Table 1. R-I of the AGAs typically stacks with G1491, although the interactions of the rRNA with R-I depend on the functional groups on the ring, which may vary. For example, ABK has no functional groups at C3 and C4 (Fig. 1a), hence makes hydrogen bonds only to nucleotides A1408 and G1494. In comparison, AMK, PAR, and NEO feature active groups distributed throughout R-I, whereas PLA, despite lacking functional groups at C3 and C4, possesses a long hydroxyethyl group at N6 in R-I (Fig. 1a), which enables interaction with A1913 of the large ribosomal subunit RNA. Compared to R-I, R-II interactions are fairly conserved in all AGAs. R-III in ABK, AMK and PLA is analogous to R-IV of NEO and PAR. However, NEO and PAR have an additional ring (R-III) which together with R-IV secures extensive interactions from the neighboring bases, 1405–1407 and 1489–1491. Although R-III of ABK and AMK are chemically identical, they exhibit distinct interactions with rRNA. AMK interacts solely with G1405, whereas in our structure, ABK interacts with G1405, C1407, and G1497. Additionally, in the available *E. coli* 70S-ABK structure (PDB: 8IFC, Supplementary Fig. 4), ABK-RIII engages with G1405, U1406, C1407, U1495, C1496, and G1497. These differences may stem from variations in drug densities and modeling between the studies. ABK, PLA and AMK have a unique AHB tail at the 1-amino position of R-II, which interacts with rRNA nucleotides 1495–1498.

## Discussion

AGAs are well-known antibiotics for the inhibition of bacterial protein synthesis, yet their effects on ribosome functions are diverse. Semi-synthetic AGAs such as AMK and ABK, developed by chemical modification of natural scaffolds with the addition of bulky side chains, have been successful against several MDR bacterial pathogens^40^. In this study, we investigated how ABK and AMK (AHB-containing AGAs) vary from other aminoglycosides in terms of inhibitory activity on bacterial protein synthesis. About 6–8-fold lower MIC values for AHB-containing ABK and AMK compared to DBK and KAN revealed that ABK and AMK are significantly potent inhibitors of bacterial protein synthesis. Our in vitro fast-kinetics results closely corroborate the in vivo observation and show that ABK and AMK inhibit translocation step of bacterial protein synthesis with inhibition constant two to threefold smaller than other AGAs (Fig. 1d). Furthermore, ABK and AMK reside two to three-fold longer on the elongating ribosomes compared to their precursor AGAs as well as NEO and PAR (Fig. 1F). In addition, we observe considerable impeding effects of both ABK and AMK in the rates of RF-mediated peptide release (Fig. 2b) indicating higher potency of these antibiotics on translation inhibition in bacteria.

The cryo-EM structure of ABK bound to *E. coli* 70S ribosome in complex with mRNA and initiator tRNA determined in this study (Fig. 3) explains the higher inhibitory effects of ABK and AMK in bacterial translation inhibition. Like other AGAs^20,39^, ABK binds to h44 of 16S rRNA with the DOS ring placed in a highly similar fashion. Binding of ABK extrudes the universal monitoring nucleobases A1492 and A1493 out of their helix, thereby stabilizing the ribosomal A-site similar to an activated state of cognate codon-anticodon interactions^41^. Locking the ribosomal A-site in this conformation promotes the characteristic ‘30S-domain closure’ and subsequent activation of GTP hydrolysis on EF-Tu leads to the accommodation of non-cognate tRNAs in the ribosomal A-site^4,41,42^. This severely impairs the accuracy of mRNA translation and induces a massive influx of erroneous amino acids in synthesized proteins, as observed in our earlier study^11^. The extrahelical conformation of monitoring nucleobases further stabilizes the peptidyl tRNAs in the A-site that hinders the migration of tRNAs from the A-site to the P-site during mRNA-tRNA translocation. Furthermore, because bound ABK occupies the in-helix space of monitoring nucleobases, it inhibits the disruption of the codon-anticodon duplex following the insertion of domain-IV of EF-G into the A-site, which is essential for ribosomal translocation^43–45^ (Supplementary Fig. 5).

The observed effects of ABK and other AGAs on peptide release may also stem from a similar structural rearrangement of monitoring nucleobases. The extrahelical conformation of A1493 could clash with the domain II of class I RFs thereby impeding the binding of RF to the A-site and stop-codon recognition^46^ (Supplementary Fig. 6). This effect appears to be very short-lived for classic AGAs including KAN and DBK, as they did not show any considerable effects in our peptide release experiments (Fig. 2). Notably, the effects of AMK on peptide release are the most distinct among all AGAs. Although the reason is unclear, the additional effects of AMK most likely stem from its additional binding sites on the ribosome. Moreover, as we did not see ABK at the H69 of 23S rRNA, the modest effects of ABK in termination as observed in our earlier study could represent its allosteric effects since h44 and H69 are linked^14^. However, these require further validation from structural and functional investigations.

The observation that AHB-containing semisynthetic AGAs ABK and AMK reside longer on the elongating ribosomes compared to their precursors KAN and DBK can be easily explained by our structural findings. Although KAN, DBK, AMK, and ABK belong to the same class of AGAs (4,6-disubstituted DOS), the additional AHB moiety at the N^1^ position of RII in ABK and AMK secures additional interactions from h44 nucleotides, 1494–1498 ^30^. These interactions of the AHB moiety of ABK and AMK, in turn, stabilize these drugs at the binding pocket. As EF-G-driven dissociation of ABK occurs through the flipping-in of extruded monitoring bases A1492 and A1493 ^11,45^, additional stabilization by AHB would delay ABK dissociation from the binding pocket. Therefore, even in the presence of high EF-G concentrations, ABK (and AMK) prolong the meantime of elongation to several seconds, substantially longer than their precursor AGAs (Fig. 1F). The stable binding is also reflected in the relatively smaller K_I_ values of ABK and AMK for the inhibition of translocation (Fig. 1d). However, we also observe that the dwelling time of ABK and AMK on the ribosome is higher than the 4,6-disubstituted DOS, PAR, and NEO. The latter have a four-ring structure which shows additional interactions with h44 nucleotides, 1489–1491. Comparison of structures of these drug-ribosome complexes reveals that the number of interactions between h44 and ABK, AMK, PLA, NEO, and PAR is comparable. Nevertheless, ABK, AMK and PLA molecules have a smaller surface area compared to PAR and NEO. When a drug molecule binds to its binding pocket on the target, it incurs an entropic penalty (ΔS) due to the loss of translational, rotational, and conformational freedom. This entropy loss is generally greater for larger molecules, which have more degrees of freedom that become restricted upon binding. Therefore, even if two drugs form similar enthalpic interactions (ΔH), the overall Gibbs free energy change (ΔG = ΔH – TΔS) is often more favorable for the smaller molecule, owing to its lower entropy cost. As a result, the smaller drug may exhibit a longer dwell time on the ribosome because its binding is thermodynamically more favorable. This might explain why a similar number of interactions stabilize the ABK and AMK more than PAR and NEO, leading to a longer dwelling time on the ribosome.

In summary, our structural and biochemical findings coupled with analysis of other published data suggest that additional interactions of AHB moiety with rRNA nucleobases provide added stability and longer dwelling time for ABK and AMK, which is indeed the basis for their enhanced inhibitory effect on bacterial ribosomes. This echoes the lower in vivo minimum inhibitory concentration (MIC) of ABK against multiple bacterial pathogens^47^. ABK, owing to modifications in the AME target sites not only evade the action of multiple AMEs but also impart potent inhibitory effects. The long dwelling time of ABK on the translating ribosome most likely leads to longer post-antibiotic effects of ABK during treatment^16,48^. Altogether, our findings clarify the rationale behind the potent effects of new-generation AGAs which would strengthen the efforts toward the design of future antibiotics.

## Materials and methods

### Buffers and components

All experiments were carried out in HEPES-polymix buffer (pH 7.5) at 37 °C supplied with energy regeneration components such as 1 mM ATP, 1 mM GTP, 10 mM phosphoenolpyruvate (PEP), 50 µg/mL pyruvate kinase (PK), 2 µg/mL myokinase (MK) ensuring a physiological range of the free Mg^2+^ concentration. 70S ribosomes were prepared from JE-28 *E. coli* following standard procedures^49^. All others translation components were prepared from the overexpression of in-house clones in *E. coli* BL21 (DE3) cells and purified using the Ni-IMAC method. Initiator tRNA, f[^3^H]Met-tRNA^fMet^, aminoacyl tRNAs and BODIPY™ (BOP) Met-tRNA^fMet^ were purified as described earlier^11,35,50^. XR7-mRNA with a strong Shine-Dalgarno sequence (AAGGAGG) and a small ORF sequence AUGUUCCUGUAA (Met-Phe-Leu-stop) was prepared using *in vitro* transcription. Pyrene labeled mRNA coding for Met-Phe-Leu (sequence 5′-UAACAAUAAGGGAGUAUUAAAUGUUCCUGC 3′-pyrene) was purchased from Ella Biotech, Germany. Arbekacin sulfate was from Carbosynth, the United Kingdom. All other AGAs were from sigma Aldrich. Other chemicals were either from Sigma-Aldrich or Merck.

### Kinetics of EF-G catalyzed mRNA movement during translocation

The initiation mix (IM) contained 70S ribosomes (0.5 µM), 3ʹ Pyrene-labeled mRNA coding Met-Phe-Leu (0.7 µM), IF1 (0.5 µM), IF2 (1 µM) and IF3 (0.5µM), f^3^H] Met-tRNA^fMet^ (0.6 µM), GTP (1 mM) and ATP (1 mM). The elongation mix (EM) contained EF-Tu (5 µM), EF-Ts (2 µM), EF-G (5 or 20 µM), Phenylalanine (200 µM), Phe RS (0.5 µM), tRNA^Phe^ (12 µM), GTP (1 mM) and ATP (1 mM). To check the effect of AGAs on translocation, an equal amount of each drug (0–10 µM) were added to both IM and EM. These two mixes were incubated at 37 °C for 15 min and equal volumes of IM and EM were rapidly mixed in a stopped-flow instrument (µSFM BioLogic) at 37 °C. The changes in pyrene-mRNA fluorescence due to translocation were monitored using a 360-nm long-pass filter (Comar Optics Ltd.) after exciting at 343 nm^11,34^. The resultant fluorescence traces were fitted with a double exponential function using Origin Pro 2016 (OriginLab Corp) and the rates and amplitudes of the fast and slow phases were estimated. From the slow phase mean time the dwelling time of the AGAs on the ribosome has been estimated as published earlier^11^.

### Kinetics of RF mediated peptide release

Pre-termination ribosome complex (Pre-TC) containing BODIPY™ (BOP)-Met-Phe-Leu-tripeptidyl tRNA^Leu^ in the P-site and a stop codon (UAA) in the A-site was prepared in HEPES polymix buffer (pH 7.5) as described earlier^35^. Equal volumes of pre-incubated Pre-TC (0.1 µM) and RF mix containing RF1 or RF2 (1 µM) were rapidly mixed in a stopped-flow instrument (µSFM BioLogic) at 37 °C. The release of (BOP)-Met-Phe-Leu tripeptide was followed by monitoring the decrease in BOP fluorescence (excitation: 575 nm) with a cutoff filter of 590 nm. The fluorescence traces were fitted with a double exponential function using Origin Pro 2016 (OriginLab Corp) and the rates of peptide release were estimated from the predominant fast phase as described earlier^35^. To check the effect of AGAs on peptide release, an equal amount of each drug (0–10 µM) was added to both mixes.

### Minimum inhibitory concentration (MIC) measurement

The minimum inhibitory concentrations (MIC_50_) of Arbekacin (ABK), Amikacin (AMK), Dibekacin (DBK) and Kanamycin (KAN) were determined by broth microdilution (BMD) method following Clinical and Laboratory Standard Institute (CLSI) guidelines for aminoglycosides^51^. Two-fold serial dilutions of the AGAs were prepared in cation-adjusted Mueller Hinton broth (CA-MHBII) corresponding to the concentrations ranging from 0.25 to 256 µg/mL and added to the 96-well (12 × 8) round-bottomed microtiter plate. Media alone without any antibiotic served as growth control. *E. coli* MG1655 suspensions equivalent to 5 × 10^5^ CFU/mL prepared from a single colony were added to the wells containing various AGA concentrations. The microtiter plates were incubated at 37 °C for 16 to 18 h and MIC was estimated as the lowest concentration of the drugs that prevented the visible growth of bacteria. The experiments were repeated three times.

### Cryo-EM sample and grid preparation plus imaging

A mixture of 70S ribosomes (200 nM) with ABK (8 µM) was incubated for 5 min at 37 °C. XR7 mRNA (Met-Phe-Phe-Stop) (2 µM) and fMet-tRNA^fMet^ (5 µM) were added to it and incubated for another 10 min. Quantifoil R2/2 300 mesh copper grids with a 2 nm carbon layer were used. These grids were glow discharged in a PELCO easiGlow at 20 mA for 30 s. Vitrobot Mark IV (FEI ThermoFisher) was used at 4 °C, 100% humidity, blot force 0, and blot time 3 s, for vitrification of 3 µl of the sample. The grid was imaged on a Titan Krios (FEI ThermoFisher) at 300 keV using a K2 direct electron detector at a resolution of 0.85 Å/pixel. A total of 7228 image stacks, 20 frames each were collected with a dose per frame of 1.35 e/Å^2^ (Supplementary Table 2).

### Image processing

Relion 3.1.2 was used for image processing (Supplementary Fig. 7). Motion correction was performed on the image stacks using MotionCor2^52^. The contrast transfer function parameters were estimated using CTFFIND-4.1^53^. Automatic particle picking was performed followed by 2D classification. With suitable 2D classes, reference-based particle picking was performed on all micrographs (482572 particles from 4858 micrographs). The particles were extracted in each case with 5X binning i.e. 500 px to 100 px. These particles were subjected to the second round of 2D classification and suitable class averages (67685 particles) were chosen for further processing. These particles were used for 3D refinement to generate an initial model using a 20 Å low pass-filtered 70S ribosome map. The refined particles were used for 3D classification to resolve conformationally distinct states and unwanted particles. Particles from high-resolution monosome 3D classes were selected. The selected particles were unbinned (100 px to 500 px) and 3D-refined with a monosome mask. CTF refinement and particle polishing were performed on the 3D refined particles (46282) followed by another round of auto refinement and post-processing to obtain a 3.1 Å map. The gold standard Fourier shell correlation (FSC) value of 0.143 was used for evaluating the averaged map resolution. Resmap was used for estimating the local resolution^54^.

### Model building

The cryo-EM structure of *E. coli* 70S was used as a reference model (PDB: 7K00). The model was fit into the map through rigid-body fit in UCSF Chimera^55^ and Chimera X^56–58^. The model was inspected in COOT^59^ and regions of r-proteins or rRNA for which density was missing were deleted from the final model. COOT was used to improve the fit of the model to map and for manual model adjustments and editing before iterative rounds of real space refinement using PHENIX^60,61^. The model was validated using comprehensive validation within PHENIX^60,61^ and PDB OneDep (Supplementary Table 2).

## Electronic supplementary material

Below is the link to the electronic supplementary material.

Supplementary Material 1

## Data Availability

The cryo-EM map and the corresponding atomic coordinates generated in this study have been deposited in the Electron Microscopy Data Bank (EMD:48329) and the Protein Data Bank (PDB:9MKK).
