# Supplementary material for: Structure–function comparison of Arbekacin with other aminoglycosides elucidates its higher potency as bacterial translation inhibitor
Source: Sci Rep. 2025 May 25;15:18271. doi: 10.1038/s41598-025-02391-3 (PMC12104382; doi:10.1038/s41598-025-02391-3)
Supplement: Supplementary file 1 — Supplementary Material 1 [file 41598_2025_2391_MOESM1_ESM.pdf]

# Supplementary Information

## Structure-function comparison of arbekacin with other aminoglycosides elucidates its higher potency as bacterial translation inhibitor

Soneya Majumdar‡, Narayan Prasad Parajuli‡, Xueliang Ge, Suparna Sanyal*
Department of Cell and Molecular Biology, Biomedical Center, Uppsala University, SE-75124, Uppsala, Sweden

‡ Equal contribution
*Corresponding author: suparna.sanyal@icm.uu.se

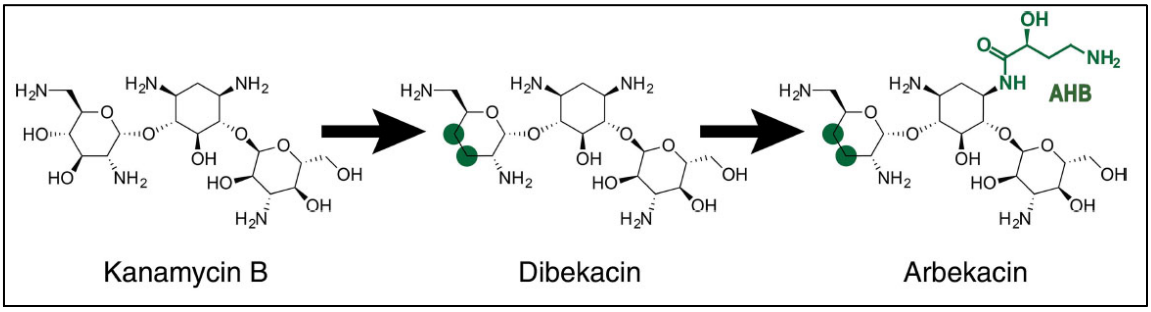


**Supplementary Figure 1. Synthesis of Arbekacin from Kanamycin via Dibekacin.**
The pathway of synthesis of Arbekacin from Dibekacin, by addition of the amino-2-hydroxybutyric moiety (AHB, in green) to it. DBK itself is derived from Kanamycin B by replacing its two hydroxyl groups with hydrogen (marked with green circles). These hydroxyl groups are common targets for aminoglycoside-modifying enzymes, a key mechanism behind aminoglycoside resistance.

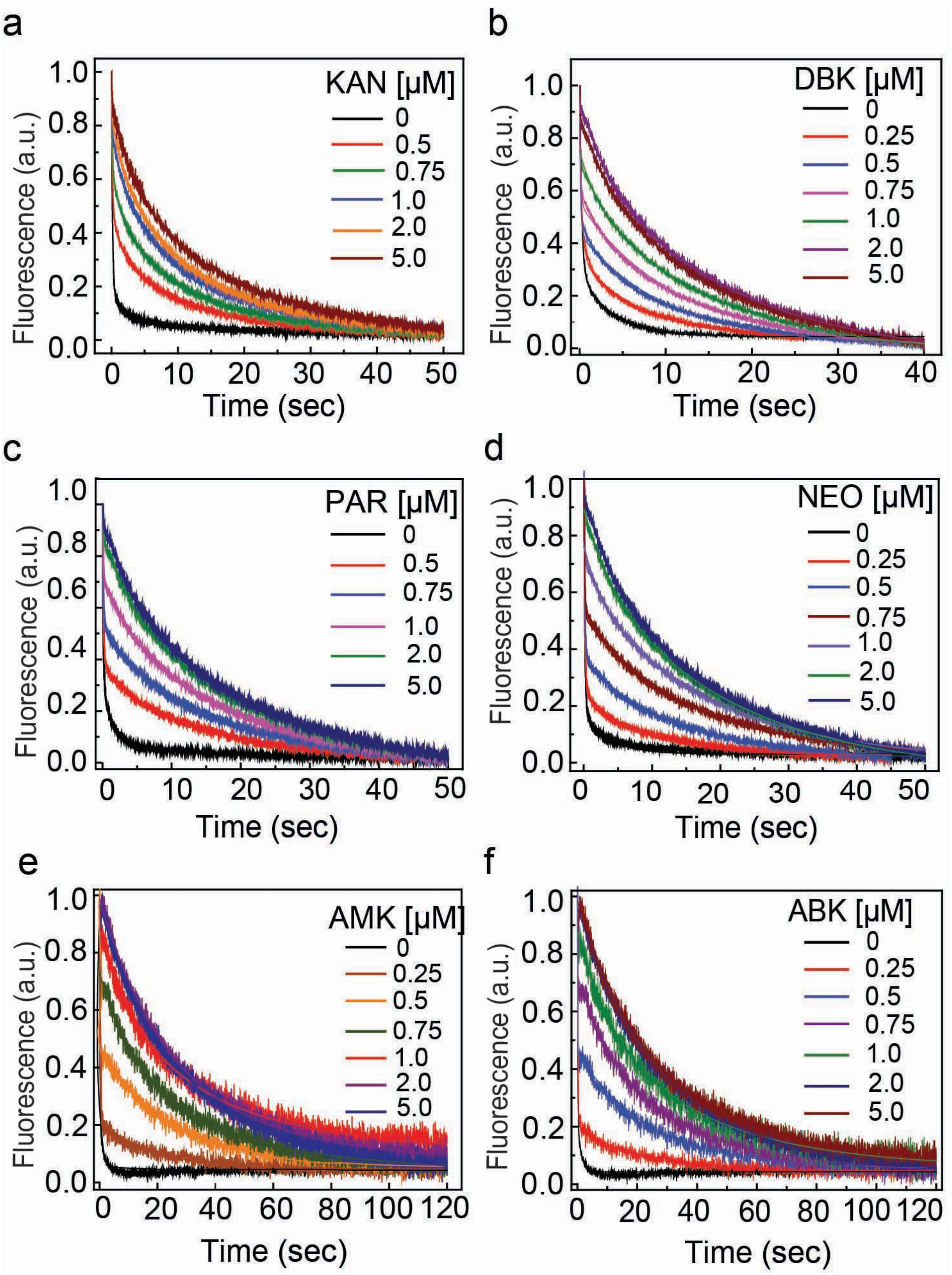


**Supplementary Figure 2: Effects of various aminoglycosides on mRNA-tRNA translocation.** Time courses of EF-G (5 μM) catalyzed translocation monitored by change in fluorescence of pyrene-labeled mRNA, in the presence of (a) Kanamycin (KAN), (b) Dibekacin (DBK), (c) Paromomycin (PAR), (d) Neomycin (NEO), (e) Amikacin (AMK), and (f) Arbekacin (ABK) added with the final concentration indicated in the figure panels.

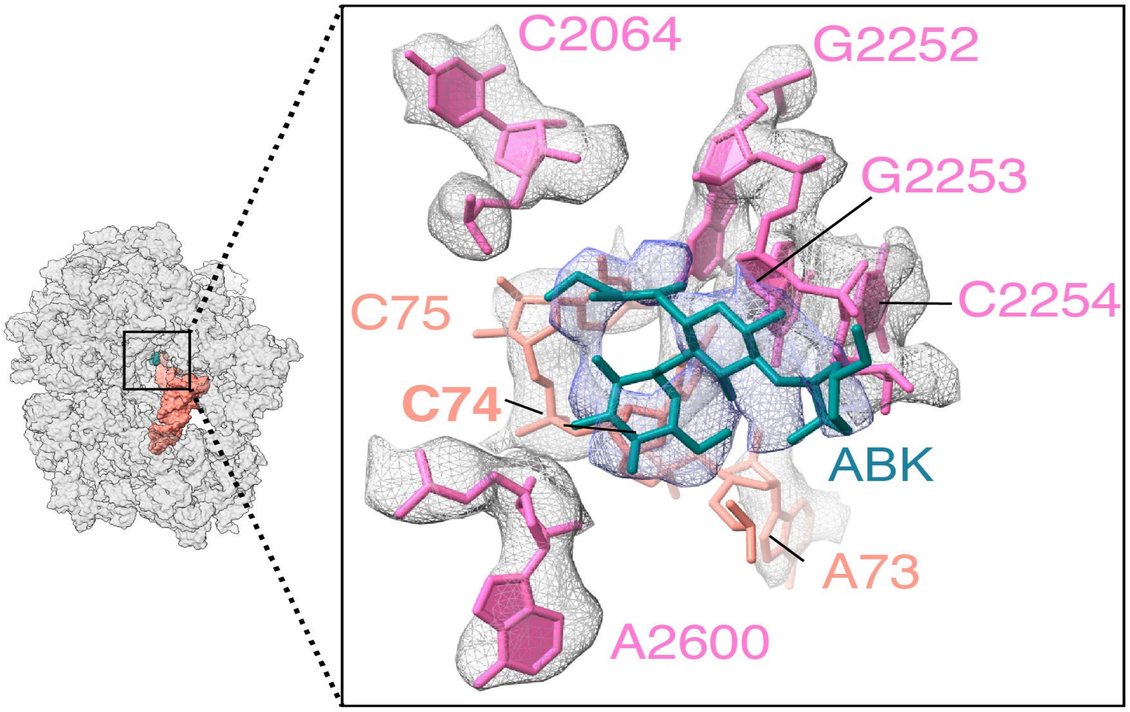


**Supplementary Figure 3.** Thumbnail showing the second ABK binding site on 50S of the *E. coli* 70S ribosome. The zoomed inset shows EM density and molecular model of ABK (teal), 23S rRNA nucleotides (pink) and P-tRNA CCA nucleotides (salmon) interacting with it. Due to poor occupancy the interactions are not highlighted.

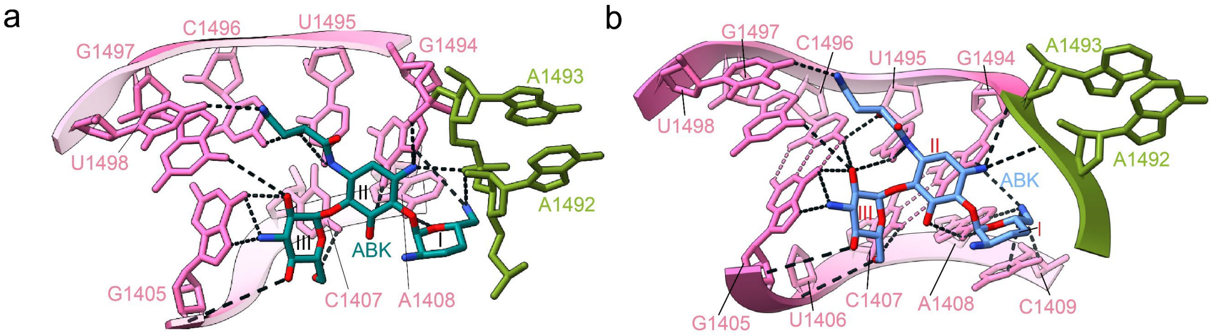


| | I | II | III | AHB |
|---|---|---|---|---|
| Ec-70S-ABK (this study) | 1408, 1491, 1494 | 1493,1494 | 1405, 1407, 1497 | 1495,1496, 1498 |

| | I | II | III | AHB |
|---|---|---|---|---|
| Ec-70S-ABK (PDB:8IFC) | 1408,1409, 1494 | 1408,1493, 1494 | 1405,1406,1407, 1495,1496,1497 | 1495,1496, 1498 |

**Supplementary Figure 4. Comparison of interactions stabilizing ABK in the primary binding site at h44.** (a) our structure of *E. coli* 70S-fMet-tRNA$^{fmet}$-mRNA-ABK complex (PDB:9MKK, this study), (b) *E. coli* 70S bound to A and P site tRNA and ABK (PDB: 8IFC). The monitoring bases A1492 and A1493 are shown in olive green, and the h44 nucleotides interacting with the drug are shown in pink. The interactions stabilizing RI-RIII and the AHB tail of the drug are listed in the table below each panel.

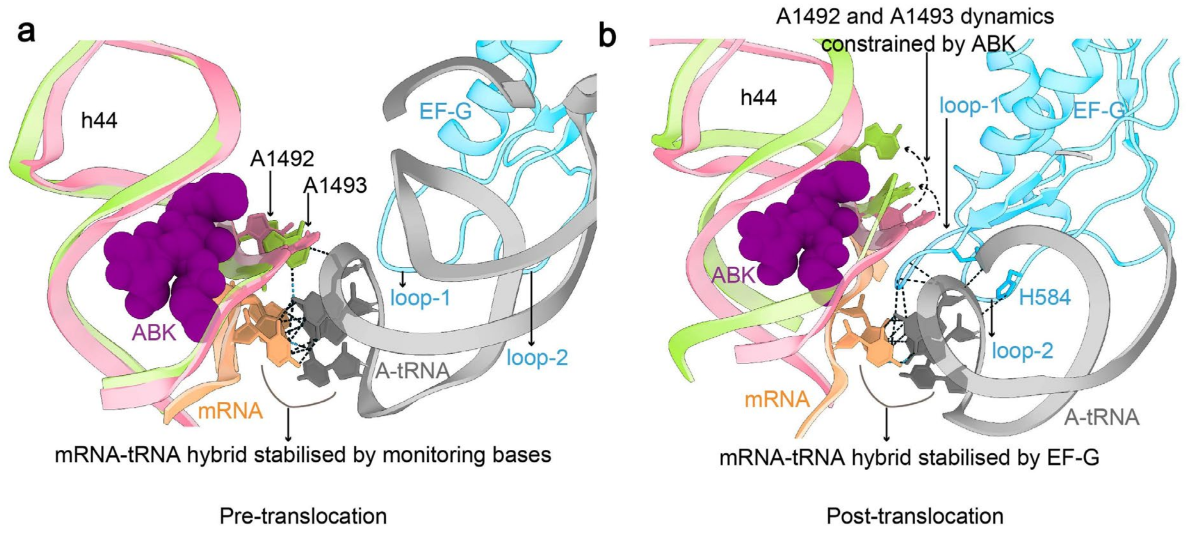


**Supplementary Figure 5. ABK binding in the decoding center and potential obstruction for EF-G catalyzed translocation.** Superposition of ABK-70S structure (this study) and EF-G bound a) pre-translocation (PDB:7SSL) and b) post-translocation ribosome (PDB: 7SSD). ABK induced flipping-out of monitoring nucleobases A1492 and A1492 lock the codon-anticodon minihelix in the decoding center of pre-translocation ribosomes, which pose a potential hurdle for the insertion of domain IV of EF-G in the decoding center for translocation of A-site tRNA.

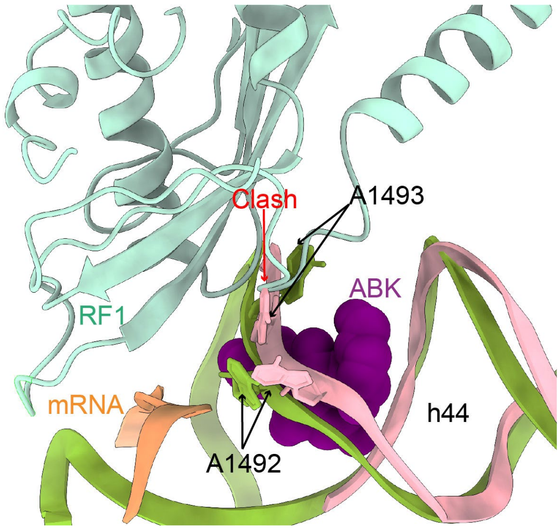


**Supplementary Figure 6. ABK binding in the decoding center and potential obstruction for RF1 binding.** Superposition of our structure with RF1 bound *E. coli* 70S ribosome (PDB: 6GWT). ABK induced extrahelical stabilization of monitoring nucleobase A1493 clashes with the domain II of RF1, which may prevent stop codon recognition and subsequent peptide release.

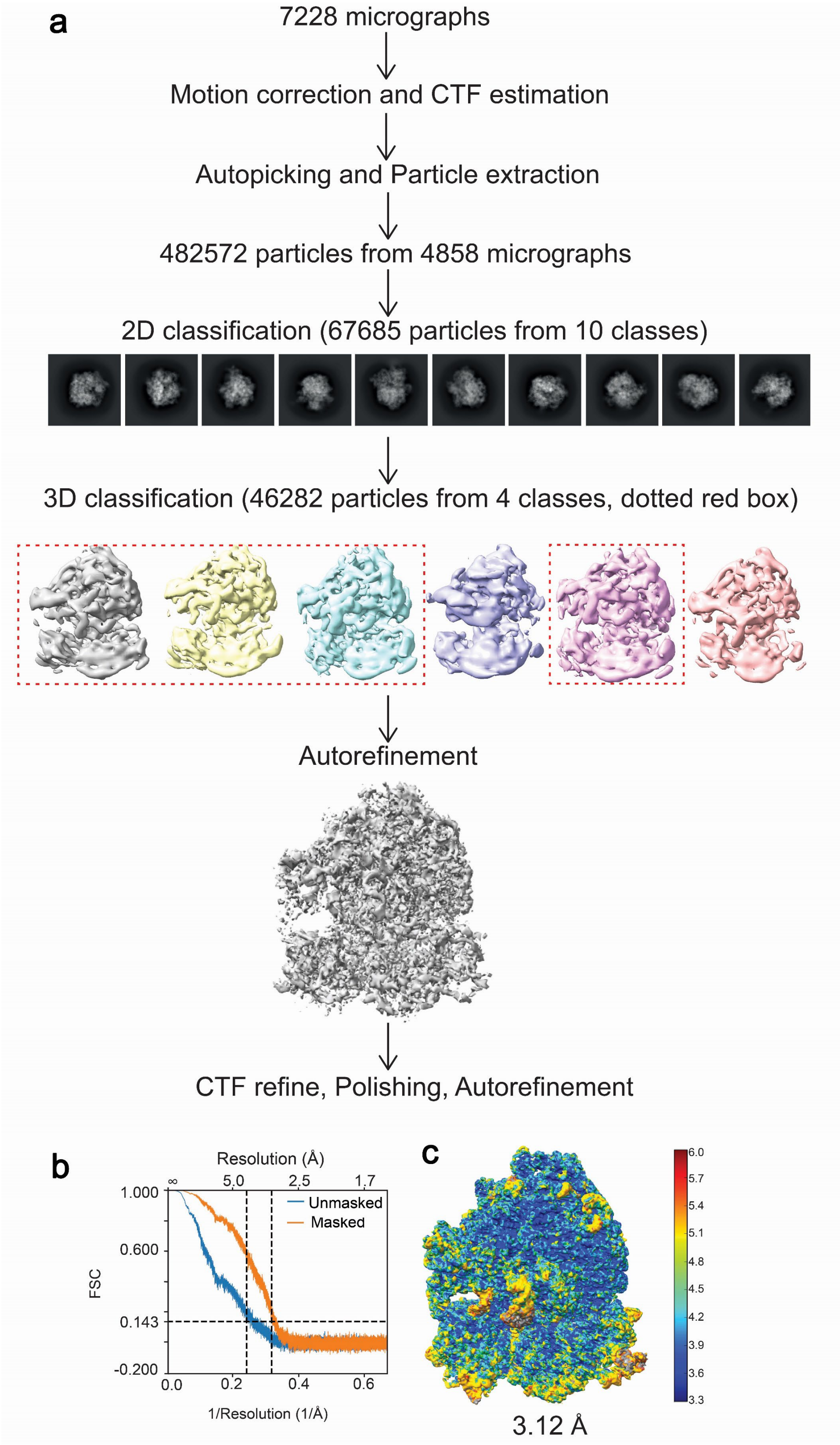


**Supplementary Figure 7. Pipeline for cryo-EM data processing using Relion 3.12.** (a) Image reconstruction workflow, (b) Fourier shell correlation (FSC) curves for half maps without (blue) and with mask (orange), (c) Local resolution distribution of the map.

**Supplementary Table 1. Interactions of various parts of aminoglycoside antibiotics with 16S rRNA nucleobases at the h44 A-site decoding center of bacterial ribosome.**

| **Ring** | **Arbekacin** | **Amikacin** | **Plazomicin** | **Paromomycin** | **Neomycin** |
|---|---|---|---|---|---|
| RI | 1408, 1491, 1493, 1494 | 1408, 1491-1492 | 1494, 1408, 1913* | 1408, 1409, 1491-1494 | 1408, 1492-1494 |
| RII | 1493, 1494 | 1408, 1493-1494 | 1408, 1493, 1494 | 1405, 1493-1495 | 1405, 1406, 1493-1495 |
| RIII/RIII+RIV | 1497, 1405, 1407 | 1405 | 1405 | 1405-1407, 1489-1491 | 1405, 1406, 1489-1491 |
| AHB moiety | 1495, 1496, 1498 | 1495-1498 | 1404, 1495-1498 | | |

*23S rRNA – a potential long-range interaction

**Supplementary Table 2. Cryo-EM data collection and refinement statistics**

| **Data collection** | |
|---|---|
| Microscope | Titan Krios |
| Camera | Gatan K3 |
| Voltage (kV) | 300 |
| Magnification | 105000 |
| Pixel size (Å/pix) | 0.85 |
| Defocus range (µm) | 1.5 - 2.5 |
| Total dose (e/Å$^2$) | 27.4 |
| Micrographs collected | 7228 |
| | |
| **Refinement** | |
| Number of particles (autopicked) | 482572 |
| Number of particles (used for 3D reconstruction) | 46282 |
| Resolution (Å; at FSC = 0.143) | 3.1 |
| CC (model to map fit) | 0.81 |
| | |
| **Model quality** | |
| Bonds (Å) | 0.007 |
| Angles (°) | 0.812 |
| | |
| **Validation** | |
| Clashscore | 7.37 |
| | |
| **Proteins** | |
| MolProbity score | 1.9 |
| Rotamer outliers (%) | 0.59 |
| Ramachandran favored (%) | 91.73 |
| Ramachandran allowed (%) | 8.19 |
| Ramachandran outliers (%) | 0.08 |
